# Actin depolymerizing factor-based nanomaterials: A novel strategy to enhance *E. mitis*-specific immunity

**DOI:** 10.3389/fimmu.2022.1080630

**Published:** 2022-12-21

**Authors:** ZhengQing Yu, LiXin Xu, Ke He, MingMin Lu, RuoFeng Yan, XiaoKai Song, XiangRui Li

**Affiliations:** ^1^ School of Agriculture, Ningxia University, Yinchuan, Ningxia, China; ^2^ Ministry of Education (MOE) Joint International Research Laboratory of Animal Health and Food Safety, College of Veterinary Medicine, Nanjing Agricultural University, Nanjing, Jiangsu, China

**Keywords:** *Eimeria mitis*, actin depolymerizing factor, nanomaterial, nanosphere, immune protection

## Abstract

The epidemic of avian coccidiosis seriously threatens the animals’ welfare and the economic gains of the poultry industry. Widespread in avian coccidiosis, *Eimeria mitis* (*E. mitis*) could obviously impair the production performance of the infected chickens. So far, few effective vaccines targeting *E. mitis* have been reported, and the nanovaccines composed of nanospheres captured our particular attention. At the present study, we construct two kinds of nanospheres carrying the recombinant *E. mitis* actin depolymerizing factor (rEmADF), then the characterization was then analyzed. After safety evaluation, the protective efficacy of rEmADF along with its nanospheres were investigated in chickens. The promoted secretions of antibodies and cytokines, as well as the enhanced percentages of CD4^+^ and CD8^+^ T cells were evaluated by the ELISA and flow cytometry assay. In addition, the absolute quantitative real-time PCR (qPCR) assay implied that vaccinations with rEmADF-entrapped nanospheres could significantly reduce the replications of *E. mitis* in feces. Compared with the rEmADF-loaded chitosan (EmADF-CS) nanospheres, the PLGA nanospheres carrying rEmADF (EmADF-PLGA nanosphers) were more effective in up-regulating weight efficiency of animals and generated equally ability in controlling *E. mitis* burdens in feces, suggesting the PLGA and CS nanospheres loaded with rEmADF were the satisfactory nanovaccines for *E. mitis* defense. Collectively, nanomaterials may be an effective antigen delivery system that could help recombinant *E. mitis* actin depolymerizing factor to enhance immunoprotections in chicken against the infections of *E. mitis*.

## 1 Introduction

As a parasitic enteric disease mainly induced by one or more *Eimeria* species, avian coccidiosis can cause malabsorption, reduction in growth, even increased mortality (1). As estimated, the property losses caused by chicken coccidiosis exceed USD 3 billion yearly (2). Normally, there is consensus that seven *Eimeria* species are considered to be infectious to the chicken (3), and these *Eimeria* species can be grouped into those leading to hemorrhagic enteritis (*E. tenella*, *E. necatrix*, and *E. brunetti*) and those leading to deficiency in nutrient’s absorption (*E. mitis*, *E. acervulina*, *E. praecox*, and *E. maxima*) (4). Interestingly, each *Eimeria* specie revealed a predilection for certain part of the chicken gut. Although it is considered to be less-pathogenic in chickens, *E. mitis* and *E. praecox* could obviously inhabit the productivity of animals (5, 6), posing a serious threat to the poultry husbandry worldwide (7, 8). According to the previously published papers, *E. mitis* has been proved to be related to low growth efficiency and poor laying performance (5, 7). Nowadays, the prevention strategies against avian coccidiosis primarily depends on the anticoccidial drugs or live attenuated vaccines (9). The sulphonamides are used as the main drugs for avian coccidiosis since 1940s, no new efficient ingredients are close to the market, except for the introduction of Diclazuril in 1990 (10). Furthermore, constraints such as the drug resistance and cost of live vaccines are of main issues that holds the progress of poultry husbandry (11). Under these circumstances, control of avian coccidiosis has become a main concern in poultry industry (11, 12).

Safe and reliable anticoccidial vaccines may be the best approach to reduce the burdens of *Eimeria* species (13, 14). Nowadays, anticoccidial vaccines have gained a considerable development, but the vaccines which could provide full protection against *Eimeria* species are still unavailable (15). As an actin-binding protein, the actin-depolymerizing factor (ADF) possesses high conservatism in eukaryotes, and could depolymerize filamentous actin to monomeric actin (16).The genomes of apicomplexan parasites contain few actin-binding proteins including ADF and which modulates motility processes of parasites (17). In addition, the movement of intracellular parasites rely on the rapid turnover of actin filaments (18), and the critical role of *E. tenella* ADF in the invasion of parasite has been proved (19). According to a previously published paper, the DNA vaccines expressing *E. tenella* ADF with 3-1E protein could improve host immunity against coccidiosis in chickens (20). These publications lent credit to the idea that a critical role played by *E. mitis* ADF (EmADF) in the invasion of avian coccidiosis, and development of anticoccidial vaccines targeting the EmADF seems to be practical in inducing robust immunity against *E. mitis* infections.

Currently, commercial vaccine strategies against avian coccidiosis are intensively concentrated in the live attenuated and inactivated vaccines (2).However, live attenuated anticoccidial vaccines allows the duplications of *Eimeria* species *in vivo* to generate adequate immunoprotections against coccidiosis, such strategy may lead to virulence recovery after vaccine immunizations (21, 22). Moreover, inactivated coccidiosis is more likely to induce strongly nonspecific immunity, and can bring on side effects (23). The occurrence of DNA and recombinant subunit vaccines can effectively make up these problems, and the two types of vaccines are proved to be effective in resisting avian coccidiosis in the previous publications (14). Even so, limitations also occurred in DNA vaccines as the risk of foreign DNA integration in host genomes (23), while the recombinant subunit vaccines are easy to be degraded by enzyme *in vivo* (24). Collectively, an effective vaccine resisting avian coccidiosis is still unavailable. Recently, nanomaterials served as the biodegradable delivery system have appeared in vaccines (25, 26), it can prevent antigens from undesirable degradation and enhance the immunogenic characteristics of entrapped antigens (27, 28). As a bio-based and fully biodegradable polymer, poly lactic-co-glycolic acid (PLGA) has been licensed by FDA and EMA in the manufacturing process of vaccines and drugs (29). With the nature characteristics of biocompatibility, biodegradability, and non-toxicity, PLGA has been widely used in vaccine synthesis and considered to be effective in antigen delivery (30). However, PLGA also exhibited many weaknesses in antigen delivery, when loading negative molecules. Chitosan (CS), a cationic polysaccharide, could solve this problem. CS is an attractive nanomaterial because of its good biocompatibility, biodegradable, and non-toxic (31, 32), and is proved to be safe in wound dressings and biomedical materials, even in partial food industry (33).

Followed by the views mentioned, the *E. mitis* actin depolymerizing factor (EmADF) was first expressed by prokaryotic expression system, the obtained recombinant EmADF protein (rEmADF) was then entrapped in PLGA and CS to synthesize the nanovaccines (EmADF-PLGA and EmADF-CS nanospheres). The immunoprotections of synthesized nanospheres was investigated in chickens. These observations highlighted the novel nanospheres in inducing *E. mitis*-specific immunity, and it should be an effective strategy with high-priority to prevent *E. mitis*.

## 2 Materials and methods

### 2.1 Animals and parasites

Used to provide a relevant model in this study, newborn Hy-Line (breed W-36) chickens were purchased from Tegeili Hatchery, Nanjing, China. All chickens were kept in a coccidia-free condition without administration of any coccidia vaccine, and had free access to sterilized food and clean water without anti-coccidia drugs. The specific pathogen-free (SPF) BALB/c mice (weighed 18-22 g) were obtained from the Model Animal Research Center, Nanjing University, Nanjing, China, and were reared in the isolators under the room temperature. The management of the animals, test operations, and sample gathering in the study were strictly followed the Ethics Procedures and Guidelines of the People’s Republic of China, and were supervised by the Animal Ethics Committee, Nanjing Agriculture University, Nanjing, China.

The purified *E. mitis* oocysts were stored in 2.5% potassium dichromate at 4°C at the MOE Joint International Research Laboratory of Animal Health and Food Safety, College of Veterinary Medicine, Nanjing Agricultural University, Nanjing, China. Followed the instructions of the previous paper (34), the *E. mitis* oocysts were large-scaled propagated, accumulated, and sporulated ten days prior to animals infections.

### 2.2 Cloning, expression and purification of recombinant EmADF protein

Based on the introductions of Trizol^®^ reagent (Vazyme Biotech Co., Ltd, Nanjing, China), extraction of 10^7^ purified *E. mitis* oocysts RNA were conducted, and cDNA was synthesized by using the reverse transcription kit (Vazyme Biotech Co., Ltd, Nanjing, China). The conserved domain sequences (CDS) of EmADF (GeneBank: XM_013496771.1) was amplified from the obtained cDNA using the primers as follows. The forward primers, 5’- CGC GGATCC ATGGCGAGCGGAATGC-3’, and the reverse primers, 5’- CCC AAGCTT TTAGGTAAGCACGCTGAGGTC-3’. High-Fidelity Master Mix (Tsingke Biological Technology, Nanjing, China) was used for PCR reaction with the recommended protocol in the instructions. The PCR products were purified by the Gel Extraction Kit (Omega Bio-Tek, Norcross, GA, USA), digested by *Bam*HI and *Hind*III restriction endonuclease (Takara Biotechnology, Dalian, China), and subcloned to a linearized pET-32a prokaryotic vector (Invitrogen Biotechnology, Carlsbad, CA, USA) by using the DNA Ligation Kit (Takara Biotechnology, Dalian, China). Then the recombinant plasmid was transferred into the *Escherichia coli* (*E. coli*) BL21 (DE3) cells (Tsingke Biological Technology, Nanjing, China), and propagated in Luria Bertani (LB) medium containing 100 μg/ml ampicillin. A Plasmid Mini Kit (Omega Bio-Tek, Norcross, GA, USA) was used to extract the recombinant plasmid, then double restriction enzyme digestion and the ABI PRISM™ 3730 XL DNA Analyzer (Applied Biosystems, Waltham, MA, USA) were conducted to determine the recombinant plasmid. After sequencing of the recombinant plasmid, the sequence analysis was conducted by the online Blast program (https://blast.ncbi.nlm.nih.gov/Blast.cgi).

The expression and purification procedures for recombinant EmADF (rEmADF) were carried out by a chelating column (HisTrap™ FF, Cytiva, Marlborough, MA, USA) following the manufacturer’s protocol. Briefly, the chemical competent cells carrying the correct plasmid were grown in LB medium containing 100 μg/ml ampicillin at 37°C (180 rpm) until the OD600 reached approximately 0.5. Induced for 4 h under the same condition with 1.0 mM isopropyl β-D-thiogalactoside (IPTG, Sigma, Saint Louis, MO, USA), the chemical competent cells were harvested and broken by supersonic technique. Then rEmADF was purified by a chelating column (HisTrap™ FF, Cytiva, Marlborough, MA, USA), and the ToxinEraser™ Endotoxin Removal Kit (GeneScript, Piscataway, NJ, USA) was used to eradicate the endotoxin. To analyze the endotoxin level and purity of rEmADF, the ToxinSensor™ Chromogenic LAL Endotoxin Assay Kit (GeneScript, Piscataway, NJ, USA) and 12% (*w*/*v*) sodium dodecyl sulfate-polyacrylamide gel electrophoresis (SDS-PAGE) were conducted. The obtained rEmADF was stored at -80°C until use. The concentrations of rEmADF were investigated by Pierce™ BCA Protein Assay Kit (Thermo Scientific, Waltham, MA, USA) before subsequent analysis.

### 2.3 Sera collections and immunoblot analysis

Negative sera were harvested from coccidia-free chickens. To obtain the positive sera against *E. mitis*, coccidia-free chickens at the age of fourteen days were first orally challenged with 5 × 10^4^ sporulated oocysts, four times in total at an interval of seven days. Seven days after the last challenge, blood samples were collected from wing vein of the challenged chickens. The collected sera were kept at -20°C until use.

Recombinant EmADF were analyzed by Western blot assays with sera to determine the recognition of rEmADF. In brief, rEmADF was first analyzed in 12% SDS-PAGE gel, subsequently transferred to polyvinylidene fluoride (PVDF) membranes (Millipore Ltd., Tullagreen, Carrigtwohill, Co. Cork, IRL) *via* the Trans-Blot Turbo (Bio-rad, Hercules, CA, USA). Then membranes were treated with TBST (tris buffered saline containing 0.5% (*v*/*v*) Tween 20) containing 5% (*w*/*v*) skimmed milk powder and incubated with chicken sera against *E. mitis* (1:100 dilutions) overnight at 4°C on a rotary shaker (30 rpm). Rinsed in TBST at room temperature for 5 min, membranes were incubated with HRP-conjugated goat anti-chicken IgY (1: 5,000 dilutions, eBioscience, San Diego, USA) for 1 h at 37°C. Finally, the proteins were visualized by Electro-Chemi Luminescence (ECL) system (Tanon, Shanghai, China). The sera harvested from coccidia-free chickens were used as a control.

### 2.4 Vaccine formulation

As published previously (35), the PLGA nanospheres were synthesized by double emulsion solvent evaporation technique (*w*/*o*/*w*) with minor alterations. Briefly, 50 mg PLGA (MW: 40,000-75,000 Da, LA/GA: 65/35, Sigma, Saint Louis, MO, USA) was first dissolved in 1.0 ml dichloromethane (DCM, Sigma, Saint Louis, MO, USA) at room temperature. 2.0 ml of 5.0% (*w*/*v*) polyvinyl alcohol (PVA, MW: 31,000-75,000 Da, Sigma, Saint Louis, MO, USA) was subsequently dropwise added. Fully mixed by a vortex at maximum speed, the liquid was kept in an ice bath and tip sonication was immediately performed in a continuous mode (durative time 2 s, interval time 2 s) under the output power of 40 W until the liquid transferred into milky white. Then 4.0 ml of rEmADF at 1.0 mg/ml concentration was dropwise added. Fully mixed again by a vortex at room temperature, the mixture was then sonicated using the same criteria mentioned above. To develop *w*/*o*/*w* emulsions, 2.0 ml of 5.0% PVA was dropwise added and tip sonication was again conducted. After passing through the 0.22 μm filtering membrane (Millipore, Billerica, MA, USA), the developed emulsions were centrifugated at 35,000 rpm for 40 min at 4°C. The supernatants were collected and stored at -20°C, and the precipitates were also collected and resuspended in double distilled water. The resuspended solution was kept at -80°C until it was fully frozen, and was completely freeze-dried (Labconco, Kansas City, MO, USA) to remove DCM. The EmADF-PLGA nanospheres were then stored at -20°C in powder form, and diluted by 1 × PBS before use.

The ionic gelation technique was utilized as described previously to synthesize chitosan nanospheres (36). To obtain 2.0 mg/ml chitosan solution, 20.0 mg of chitosan (MW: 50-190 kDa, Sigma, Saint Louis, MO, USA) was dissolved in 10.0 ml of 1.0% (*v*/*v*) aqueous solution of acetic acid, then the pH value was regulated to 5.0 by NaOH solution. Then 4.0 ml of rEmADF at 1.0 mg/ml concentration and 2.0 ml of 2.0 mg/ml sodium tripolyphosphate (TPP, Aladdin, Shanghai, China) solution were respectively dropwise mixed with 10 ml of chitosan solution through stirring. Subsequently, the mixture was kept in an ice bath, and tip sonication was conducted in a continuous mode (durative time 4 s, interval time 2 s) under the output power of 50 W for 3 min. After passing through the 0.22 μm filtering membrane, the mixture was then centrifuged at 35,000 rpm for 40 min at 4°C. The supernatants were harvested and stored at -20°C while the precipitates were dissolved in double distill water and stored at -80°C until the liquids were completely frozen. After fully freeze-dried by the same criteria mentioned above, The EmADF-CS nanospheres were stored at -20°C in powder form, and diluted by 1 × PBS before use.

### 2.5 Nanospheres characterization

To characterize the surface morphology of prepared nanospheres, EmADF-PLGA and EmADF-CS nanospheres were sent to College of science, Nanjing Agriculture University for scanning electron microscope (SEM) observation (SU8010, Hitachi, Tokyo, Japan). Nanospheres in SEM images were randomly measured by ImageJ software (version 1.8.0, NIH, Bethesda, MD, USA) to access the average diameter of prepared nanospheres. The loading capacity (LC) and encapsulation efficiency (EE) of rEmADF were investigated as described previously with slight modification (37), the concentration of uncombined proteins in the supernatant collected in section 2.4 were evaluated by BCA method, and the total volume of collected supernatant was also measured. Then LC and EE can be calculated based on the Formula (2) and (3).


(1) Uncombined protein (mg)=uncombined protein concentration × Supernatant volume

(2) $$\text{LC }\left(\%\right)=\frac{\text{Weight of nanospheres }-\text{ uncombined protein}}{\text{Weight of nanospheres}}\;\times \;100\%$$

(3) $$\text{EE }\left(\%\right)=\frac{\text{Total protein}-\text{uncombined protein}}{\text{Total protein}}\;\times \;100\%$$

The *in vitro* cumulative release of antigen from synthesized nanospheres was determined by the previously described method with minor modification (38). Nanospheres in power form were first dissolved in 1 × PBS (pH7.4) at placed in a shaker (37°C, 180 rpm). At an interval of 12 h, samples were taken out and centrifuged at 15,000 rpm for 10 min, and 20 μl of supernatant was harvested and kept at -20°C until use. Samples were resuspended and replaced, and the total volume of nanosphere solutions was recorded. After the last collection, the amount of free antigen in the supernatant was evaluated by Pierce™ BCA Protein Assay Kit. The *in vitro* cumulative release (CR) profile was evaluated by Formula (4).


(4) $$\text{CR }\left(\%\right)=\frac{\text{Total volume }\times \text{ Protein concentration}}{\text{Total loaded proteins}}\;\times \;100\%$$

To analyze the toxicity of formulated nanospheres, BALB/c mice were randomly divided into seven groups with five replicates in each group: Blank (vaccinated with equal volume of 1 × PBS), Control (vaccinated with pET-32a vector protein), EmADF (vaccinated with rEmADF), EmADF-PLGA (vaccinated with EmADF-PLGA nanospheres), and EmADF-CS (vaccinated with EmADF-CS nanospheres) groups. Each animal was intramuscularly injected with a dose containing 300 μg of antigen, and the dosage was three times the usual (5 mg/kg body weight). A booster immunization was also carried out by using the same strategy three days later. One day after the booster immunization, sera were collected from mice’s eye sockets, and the levels of blood urea nitrogen (BUN) and creatinine (Cr) were detected by the commercially available kits (Solarbio, Beijing, China). Throughout the process, the clinical status of animals was kept under constant surveillance.

### 2.6 Animal immunization and challenge

Newborn chickens were randomly allocated into seven groups with forty replicates in each group. Fourteen-day-old chickens were immunized in the leg muscles with multipoint, and the maximum dosage for single immunization was controlled within 500 μl. Detailed administration dosages were shown in **Table 1**. Seven days later, primary immunization was followed by a booster dose of immunization using the same vaccination strategy as referenced to the previous studies (39, 40). To demonstrate the protective efficacy elicited by the nanospheres, ten chickens of 22 days old from each group were orally challenged with 5 × 10^4^ *E. mitis* sporulated oocysts (high-dose challenge), while another ten chickens at the same age were orally challenged with 3 × 10^3^ *E. mitis* sporulated oocysts (low-dose challenge, **Table 1**). Seven days later, all animals were sacrificed under the supervision of Animal Ethics Committee, Nanjing Agriculture University, China. During the seven days after challenge, feces excreted by each low-dose challenged chicken were collected, fully mixed, and kept at 4°C. To investigate the body weight changes, ten of high-dose challenged chickens from each group were weighted at age of 14 day (one day before the first vaccination), 28 days (seven days after the booster vaccination), and 35 days (seven days after the challenge), and the body weight changes of each chicken were calculated following the Formula (5).


(5) $$\text{Coefficient of growth }\left(\%\right)=\frac{\text{Final weight}-\text{Initial weight}}{\text{Initial weight}}\;\times \;100\%$$

**Table 1:** Group assignment and immune procedure.

Group	Treatment (each chicken)	Time for vaccination	Infection dose (each chicken)
Blank (PBS)	Equal volume of 1 × PBS	At 15 and 22 days old	Equal volume of PBS (0 oocyst) at 29 days old
Blank (Coccidia)	Equal volume of 1 × PBS		For high-dose challenge: 5 × 10^4^ oocysts of purified *E. mitis* for each chicken at 29 days old; for low-dose challenge: 3 × 10^3^ oocysts of purified *E. mitis* for each chicken at 29 days old
Control	200 μg pET-32a vector protein		
PLGA	Equal volume of PLGA nanosphere loading 1 × PBS		
CS	Equal volume of CS nanosphere loading 1 × PBS		
EmADF	200 μg rEmADF		
EmADF-PLGA	EmADF-PLGA nanospheres containing 200 μg rEmADF		
EmADF-CS	EmADF-CS nanospheres containing 200 μg rEmADF		

### 2.7 Detection of antibody and cytokine secretion

At age of 15 (before first vaccination), 22 (seven days after the first vaccination), and 29 day (seven days after the booster vaccination and before challenge), chickens were anesthetized and sera were collected from the heart, and the sera were kept at -20°C until use. According to the previous study (40), enzyme linked immunosorbent (ELISA) assays were carried out to assess the EmADF-specific serum antibody levels. In brief, the 96-well microtiter plate (Corning Costar, Cambridge, USA) was coated with rEmADF (1 μg/well) overnight at 4°C. The sera samples were diluted 1: 100 in TBST with 5% (*w*/*v*) skimmed milk powder. After being rinsed in TBST for 5 min, plates were incubated with 100 μl of the sera for 1 h at 37°C. Rinsed again in TBST, plates were incubated with 1:8,000 dilutions (100 μl each well) of the HRP-conjugated anti-chicken IgY (Abcam, Cambridge, UK) at 37°C for 1 h. After being washed in TBST, 100 μl of 3,3’,5,5’-tetramethylbenzidine (TMB, Tiangen, Beijing, China) were added to each well of the plates to develop colors at room temperature. To stop the reactions, 100 μl of 2 M newly prepared H_2_SO_4_ solutions were added to each well. The absorbance at OD450 was measured on a microplate photometer (Thermo Scientific, Waltham, USA) within 30 min. Each group involved five biological replicates, and each replication was detected once.

The concentrations of interferon-gamma (IFN-γ), interleukin (IL) 4 (IL-4), transforming growth factor (TGF) β (TGF-β), IL-6, IL-10, and IL-17 in the collected sera were analyzed by commercially available ELISA kits (Enzyme-linked Biotechnology, Shanghai, China) according to the manufacturer’s protocol. Each group involved five biological replicates, and each replication was detected once.

### 2.8 Lymphocytes proliferation assay

At the age of 22 (seven days after the first vaccination) and 29 days (seven days after the booster vaccination and before challenge), five chickens from each group were euthanized to isolate splenic lymphocytes using the commercially available separation solution (TBD, Tianjin, China) in line with the previous report (41). The splenic lymphocytes proliferation assay was conducted using newly separated lymphocytes as reported previously with slight modifications (42). Briefly, splenic lymphocytes were diluted in Dulbecco’s Modified Eagle’s Medium (DMEM, Invitrogen Biotechnology, Carlsbad, CA, USA) containing 20% (*v*/*v*) fetal bovine serum (FBS) and 20 μg/ml recombinant EmADF proteins. Then 100 μl medium containing 10^5^ cells was added into each well of a 96-well plates. Incubated for 72 h at 37°C in a 5% (*v*/*v*) CO_2_ atmosphere, each well of the plates was added with 10 μl of Cell Counting Kit 8 reagent (CCK-8, Beyotime Biotech, Shanghai, China). After a four-hour incubation according to the manufacturer’s instructions, the absorbance at OD450 was investigated by a microplate photometer. Each group involved five biological replicates, and each replication was detected once.

### 2.9 Investigation of CD4^+^ and CD8^+^ T lymphocytes

As described in section 2.8, splenic lymphocytes were collected and resuspended in 1 × PBS. To evaluate the proportions of CD4^+^ T lymphocytes subsets, 100 μl medium containing 10^6^ lymphocytes were dually stained with FITC-conjugated anti-chicken CD3 (Southern Biotech, Birmingham, AL, USA) and APC-conjugated anti-chicken CD4 (Southern Biotech, Birmingham, AL, USA) for 30 min at 4°C in the dark. As for the percentages of CD8^+^ T lymphocytes subsets, 10^6^ lymphocytes were suspended in 100 μl PBS, and stained with FITC-conjugated anti-chicken CD3 and PE-conjugated anti-chicken CD8 (Southern Biotech, Birmingham, AL, USA) by the same strategy. After being washed in 1 × PBS, cells were sorted by a flow cytometry (Beckman Coulter Inc, Brea, CA, USA), and the populations were determined by CytExpert software (Beckman Coulter Inc, Brea, CA, USA). Noticeably, fluorescence compensation was performed based on the fluorescence minus one (FMO) control before cell sorting. Each group involved five biological replicates, and each replication was detected once.

### 2.10 *E. mitis* burdens in animals

To evaluate the immune protective efficacy generated by nanospheres, 200 mg feces collected in section 2.6 were lysed to extract genomic DNA by using Stool DNA kit (Omega Bio-Tek, Norcross, GA, USA). According to the unique sequence derived from sequence characterized amplified region (SCAR) markers, absolute quantitative real-time (qPCR) was conducted to demonstrate the parasite burdens in the feces from low-dose challenged chickens (43). To construct the reference standards, the sequence of SCAR markers (GeneBank: AY571506.1) was amplified from the DNA extracts by using the following primers. The forward primer: 5’-GCAGGGCAGGCAGGGTAG-3’, and the reverse primer: 5’-GCACGGCAGGCTCAGAAA-3’. High-Fidelity Master Mix was used for PCR reaction according to the instructions. The PCR amplicons of SCAR markers were then subcloned to a linearized pMD-19T vector (Takara Biotechnology, Dalian, China). To evaluate *E. mitis* burdens, PerfectStart^®^ Green qPCR SuperMix (TransGen Biotech, Beijing, China) was used for qPCR amplification with the guidelines of instruction. Furthermore, the melt-curve analysis was also carried out at the end of amplifications, and one uniform peak of the melting curve in each reaction was determined. Each group involved ten biological replicates, and each replication was detected once.

### 2.11 Statistical analysis

Statistical analysis was evaluated by GraphPad Prism (version 8.0, GraphPad Software, San Diego, CA, USA). Groups were evaluated by using a one-way analysis of variance (ANOVA) followed by Dunnett’s test. Comparisons among EmADF, EmADF-PLGA, and EmADF-CS group were conducted by ANOVA following Bonferroni’s correction. Values were presented as mean ± standard deviation (SD), and significance was considered at *p<* 0.05.

## 3 Results

### 3.1 Production of rEmADF and immunoblot analysis

**Figure 1:**
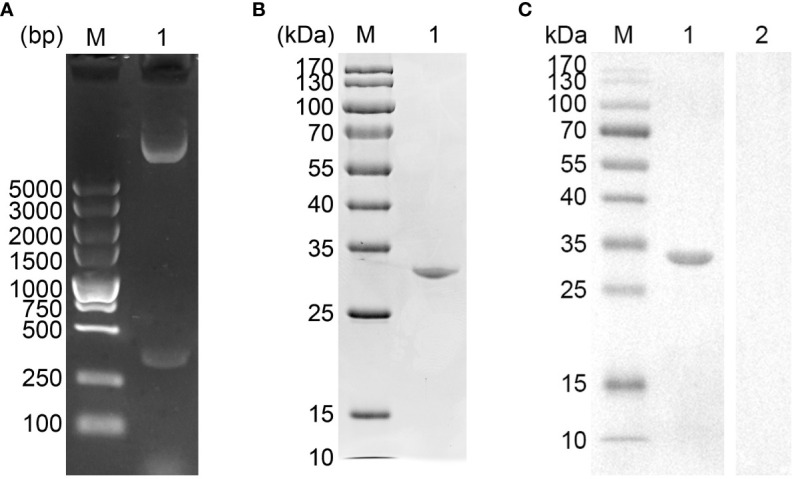
**(A)** Double digestion analysis. Line M: DL5,000 marker; Line 1: Double digestion of the constructed pET-32a-EmADF vector by *Bam*HI and *Hind*III. **(B)** SDS-PAGE analysis. Line M: MW marker proteins. Line 1: purified recombinant EmADF proteins. **(C)** Western blot analysis of purified rEmADF. Line M: MW marker proteins. Line 1: Purified rEmADF were detected by sera from *E. mitis*-infected chickens; Line 2: Purified rEmADF were detected by sera from coccidia-free chickens.

The vector of pET-32a-EmADF was successfully established, and the results of double enzyme digestion yielded two fragments, approximately 360 bp and 6,000 bp (**Figure 1A**), which was in accordance with the theory calculation (363 bp and 5,875 bp). Moreover, sequence analysis also demonstrated the pET-32a-EmADF vector was correctly constructed (**Figure S1**). Based on the guidelines of pET-32a vector, the recombinant EmADF protein expressed by the constructed vector consisted of his-tag protein (17.7 kDa) and EmADF protein (13.21 kDa). Thus, in theory, the molecular weight of the rEmADF was 30.91 kDa, which matched the result (**Figure 1B**). Furthermore, the endotoxin level in purified rEmADF felled to 0.1 EU/ml after endotoxin eradication. Demonstrated by western blot assay (**Figure 1C**), rEmADF could be identified by the chicken sera against *E. mitis*, indicating a satisfactory antigenicity of rEmADF which could elicit the host immunity.

### 3.2 Characteristics of synthesized nanospheres

**Figure 2:**
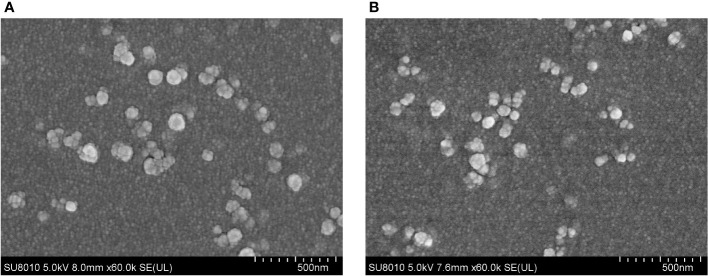
The SEM observation of EmADF-PLGA **(A)** and EmADF-CS **(B)** nanospheres. Double emulsion solvent evaporation technique was conducted to formulate EmADF-PLGA nanospheres, while the ionic technique was carried out to synthesize EmADF-CS nanospheres. Bar represented 500 nm.

The SEM results displayed that EmADF-PLGA (**Figure 2A**) and EmADF-CS nanospheres (**Figure 2B**) were uniforms, spherical, and rough surface. According to the SEM results, the diameter of EmADF-PLGA nanospheres was about 94.75 ± 10.41 nm (n = 5) in average, while the mean diameter of EmADF-CS nanospheres sized 79.55 ± 16.51 nm (n = 5). Furthermore, the LC and EE of synthesized nanospheres were also investigated. By using 5.0% PVA and 1.0 mg/ml rEmADF, the LC of EmADF-PLGA nanospheres reached 1.14% (n = 3), while the LC of EmADF-CS nanospheres reached 4.60% (n = 3) using 2.0 mg/ml TPP and 1.0 mg/ml rEmADF. According to the results of three independent trials, the EE of EmADF-PLGA and EmADF-CS nanospheres were 73.39% and 54.31%, respectively.

**Figure 3:**
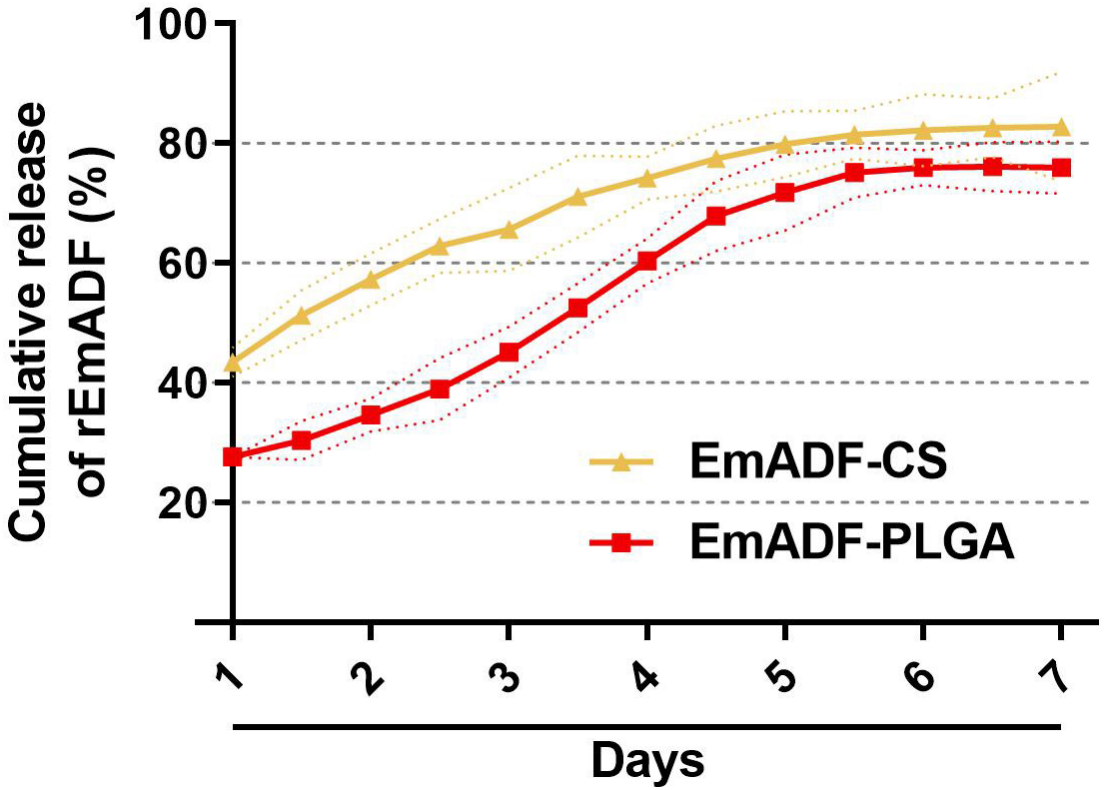
*In vitro* release of recombinant EmADF proteins from EmADF-PLGA and EmADF-CS nanospheres. The concentrations of uncombined proteins in the supernatant were investigated by BCA assay, and the CR profiles were evaluated by the concentrations and the total volumes. Three independent experiments were conducted, and each sample was measured once. Values were presented as the mean of the mean ± SD (n = 3), and SD were represented by the dotted lines.

The release profile of EmADF-PLGA and EmADF-CS nanospheres were investigated by a continuously slow release over a seven-day duration. As illustrated in **Figure 3**, a burst release indicated with around 27.61% of rEmADF was combined on the surface of PLGA nanospheres, and the initial release of EmADF-CS nanospheres demonstrated that approximately 43.48% rEmADF unbound from CS nanospheres. When compared with the Em1a-CS nanospheres within the first two days, the EmADF-PLGA nanosphere elicited a steadier release. At the fifth day, the CR curve of EmADF-PLGA nanospheres turned to be smooth, while the release profile of EmADF-CS nanospheres became flat after the fourth day.

**Figure 4:**
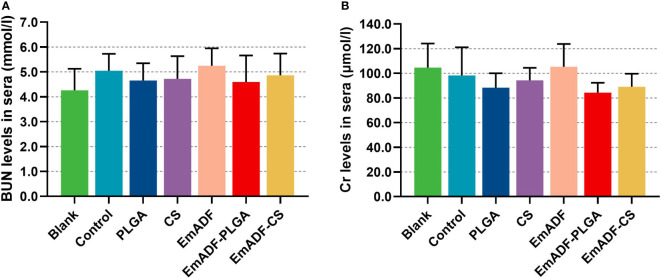
The toxicity of recombinant EmADF proteins and its nanospheres. Based on the urease-indophenol and sarcosine oxidase method, the levels of BUN **(A)** and Cr **(B)** were investigated by the commercially available kits. Each group involved five biological replicates, and each replication was detected once. Values were estimated using one-way ANOVA analysis followed by Dunnett’s test. Comparisons among EmADF, EmADF-PLGA, and EmADF-CS group were conducted by ANOVA following Bonferroni’s correction. Values were presented as the mean of the mean ± SD.

As the main point of preclinical research, the repeated dose toxicity test plays an important role in evaluating the safety of vaccines before clinical experiments. Thus, the toxicity of EmADF-PLGA and EmADF-CS nanospheres against mice was evaluated (**Figure 4**), and the levels of BUN and Cr in animals maintained in consistent with the control groups (*p* > 0.05), evaluating the health status of animals could not be affected by the synthesized nanospheres. Moreover, all mice were in good state of clinical health without adverse reaction after immunizations. All these obtained findings indicated that the recombinant EmADF and its nanospheres were harmless to the health of animals.

### 3.3 Level of antibodies and cytokines

**Figure 5:**
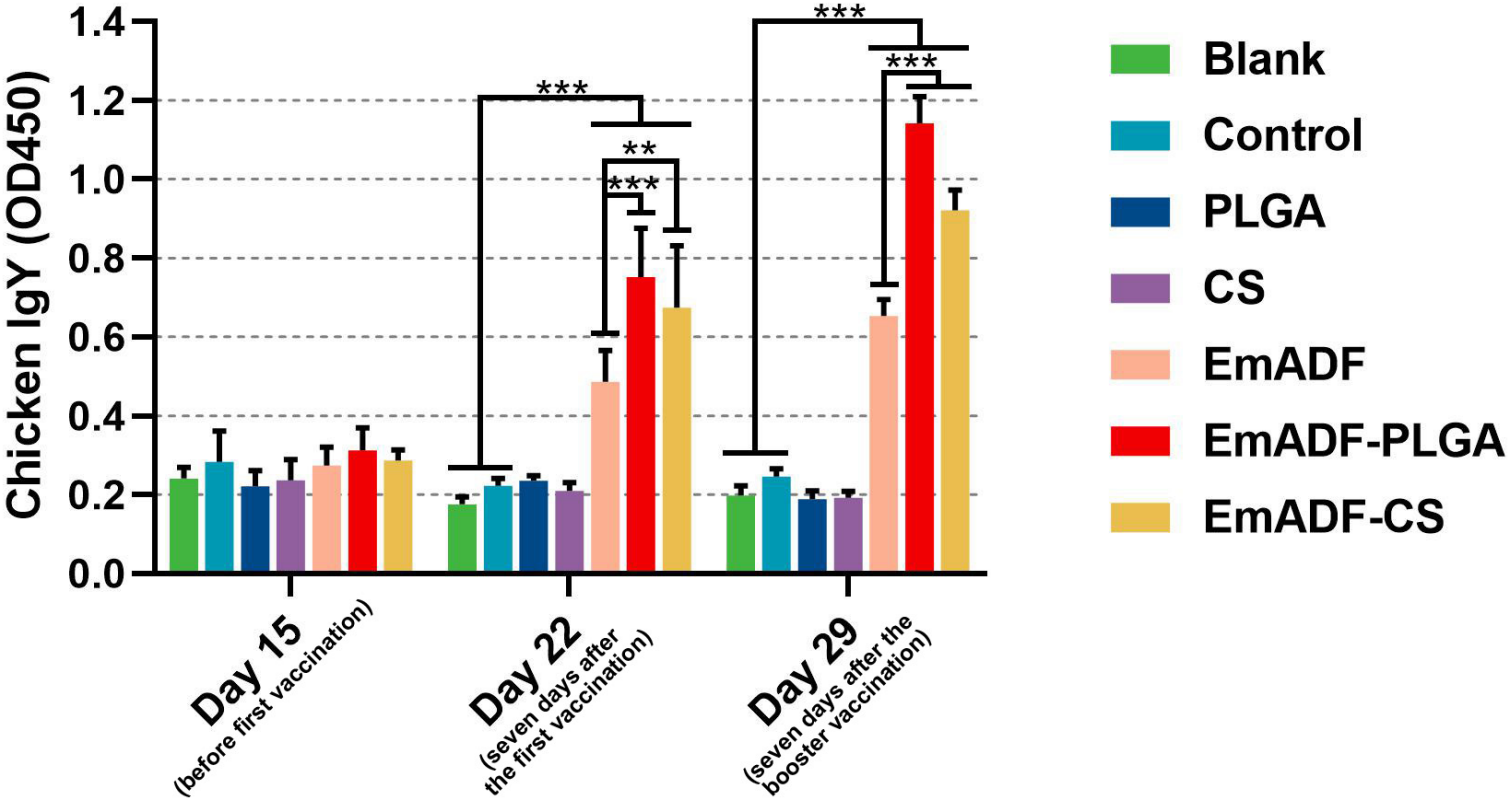
Determination of EmADF-specific antibody in the chickens’ sera. Each group involved five biological replicates, and each replication was detected once. Values were estimated using one-way ANOVA analysis followed by Dunnett’s test. Comparisons among EmADF, EmADF-PLGA, and EmADF-CS group were conducted by ANOVA following Bonferroni’s correction. Values were presented as the mean of the mean ± SD. **p< 0.01 and ***p< 0.001.

To determine the humoral immunity induced by nanospheres, animals were immunized with 200 μg of purified rEmADF or the formulated nanospheres containing equivalent rEmADF. Sera were collected at age of 15 (before the first immunization), 22 (seven days after the first immunization), and 29 day (seven days after the booster immunization), and the capacity of nanospheres to potentiate antibody immunity in chickens was evaluated by quantifying rEmADF-specific antibodies *via* standard ELISA. As illustrated in **Figure 5**, animals in EmADF, EmADF-PLGA, and EmADF-CS secreted remarkably higher levels of IgY when compared with the animals in negative groups seven days after the first and booster immunizations (*p*< 0.001). Furthermore, immunizations with EmADF-PLGA and EmADF-CS generated significantly higher IgY than that of rEmADF immunized animals at the age of 22 (seven days after the first immunization) and 29 day (seven days after the booster immunization, *p*< 0.01). When compared with the blank or control group, no rEmADF-specific antibody was detected in the chickens from the PLGA and CS group (*p* > 0.05).

**Figure 6:**
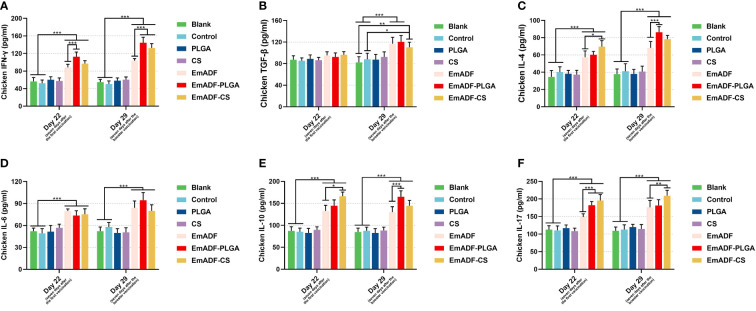
Determination of cytokines in chickens’ sera. The concentrations of IFN-γ **(A)**, TGF-β **(B)**, IL-4 **(C)**, IL-6 **(D)**, IL-10 **(E)**, and IL-17 **(F)** were illustrated by commercial ELISA kits. Each group involved five biological replicates, and each replication was detected once. Values were estimated using one-way ANOVA analysis followed by Dunnett’s test. Comparisons among EmADF, EmADF-PLGA, and EmADF-CS group were conducted by ANOVA following Bonferroni’s correction. Values were presented as the mean of the mean ± SD. *p< 0.05, **p< 0.01, and ***p< 0.001.

To further detect the results of T lymphocytes activation, sera were collected from five chickens and the secretions of cytokines were determined by the double-antibody sandwich ELISA. As exhibited in **Figure 6A**, statistically higher levels of IFN-γ could be detected in EmADF, EmADF-PLGA, and EmADF-CS group (*p*< 0.001), when compared with the negative group. Furthermore, sera from the EmADF-PLGA and EmADF-CS group showed significantly higher levels of IFN-γ than those from EmADF group (*p*< 0.001). As for the TGF-β illustrated in **Figure 6B**, no statistical difference was indicated between the rEmADF-loaded nanospheres and the EmADF group (*p* > 0.05) at the age of 22 day (seven days after the first immunization). However, the secretions of TGF-β in animals immunized with rEmADF and its nanospheres were promoted after the booster immunization (*p*< 0.05). Chickens from the rEmADF-loaded nanospheres could induce statistically higher levels of IL-4 after the first and booster immunizations (*p*< 0.001, **Figure 6C**), while at the age of 29 day (seven days after the booster immunization), sera isolated from EmADF-PLGA group were detected with significantly higher levels of IL-4 than those from the EmADF group (*p*< 0.001). Furthermore, rEmADF and its nanospheres could statistically up-regulate the secretions of IL-6 (**Figure 6D**), IL-10 (**Figure 6E**), and IL-17 (**Figure 6F**) after the first and booster immunizations. After the booster vaccination, EmADF-PLGA nanospheres could stimulate the secretions of IL-10 in chickens (*p*< 0.001, **Figure 6E**), while EmADF-CS nanospheres increased the secretions of IL-17 (*p*< 0.01, **Figure 6F**). Noticeably, comparisons between the blank and control group revealed consistency in statistics (*p* > 0.05).

### 3.4 The proliferation of lymphocytes

**Figure 7:**
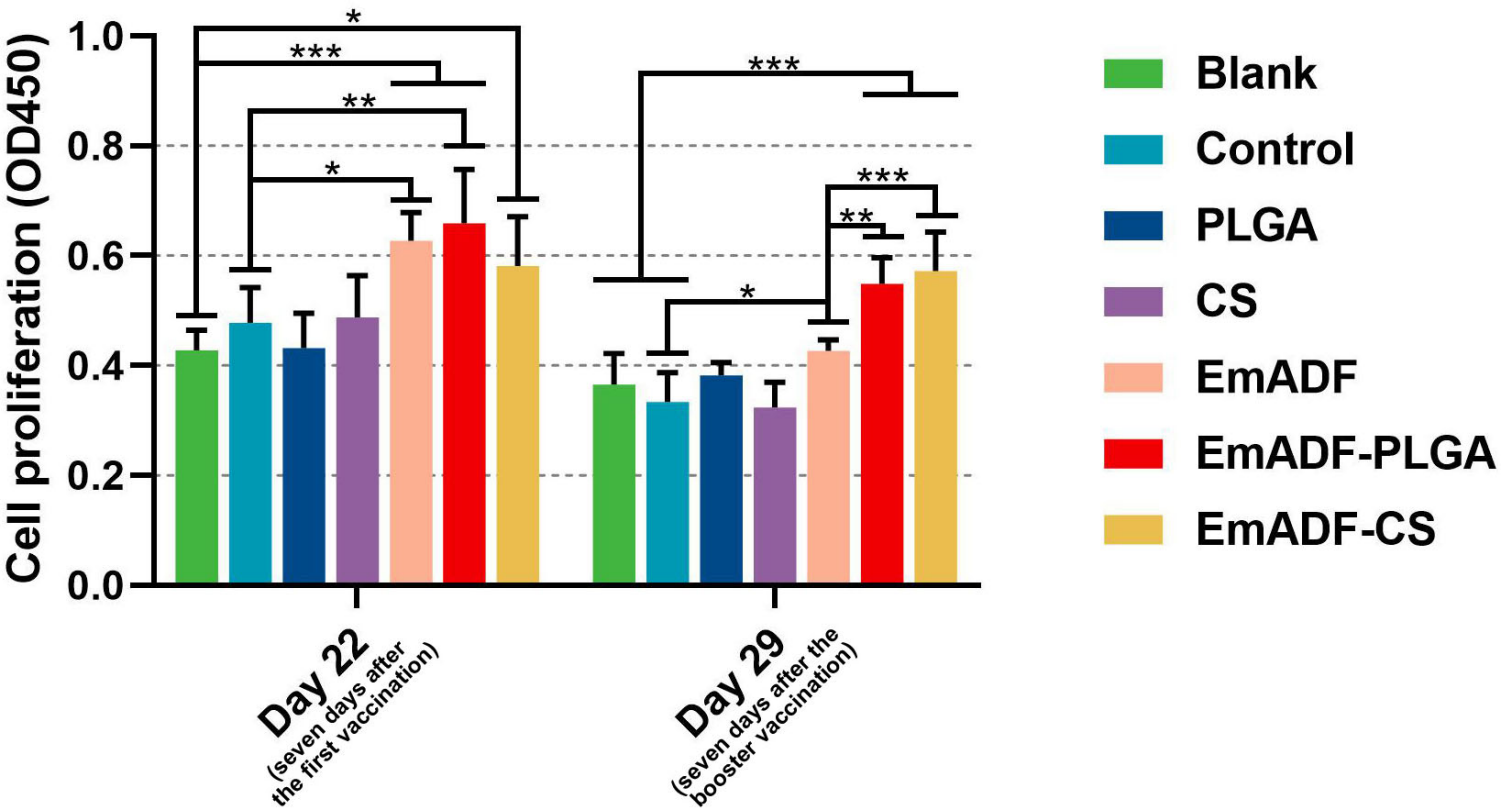
The proliferation of chickens’ lymphocytes. Chickens in each group were sacrificed, and the lymphocytes was collected. The proliferation of lymphocytes was then determined by CCK-8 reagent. Each group involved five biological replicates, and each replication was detected once. Values were estimated using one-way ANOVA analysis followed by Dunnett’s test. Comparisons among EmADF, EmADF-PLGA, and EmADF-CS group were conducted by ANOVA following Bonferroni’s correction. Values were presented as the mean of the mean ± SD. *p< 0.05, **p< 0.01, and ***p< 0.001.

The matured dendritic cells could activate T lymphocytes, then the activated T lymphocytes initiated cell proliferation. Thus, the effects of rEmADF and its nanospheres in promoting T cell proliferation was investigated. As illustrated in **Figure 7**, vaccinations of EmADF, EmADF-PLGA nanospheres, and EmADF-CS nanospheres promoted the lymphocyte proliferation when compared with the negative groups (*p*< 0.05). In addition, the promoting effects were also evaluated in EmADF-PLGA and EmADF-CS nanospheres when compared with rEmADF at the age of 29 day (seven days after the booster immunization, *p*< 0.01).

### 3.5 Identification the proportions of CD4^+^ and CD8^+^ T lymphocytes

**Figure 8:**
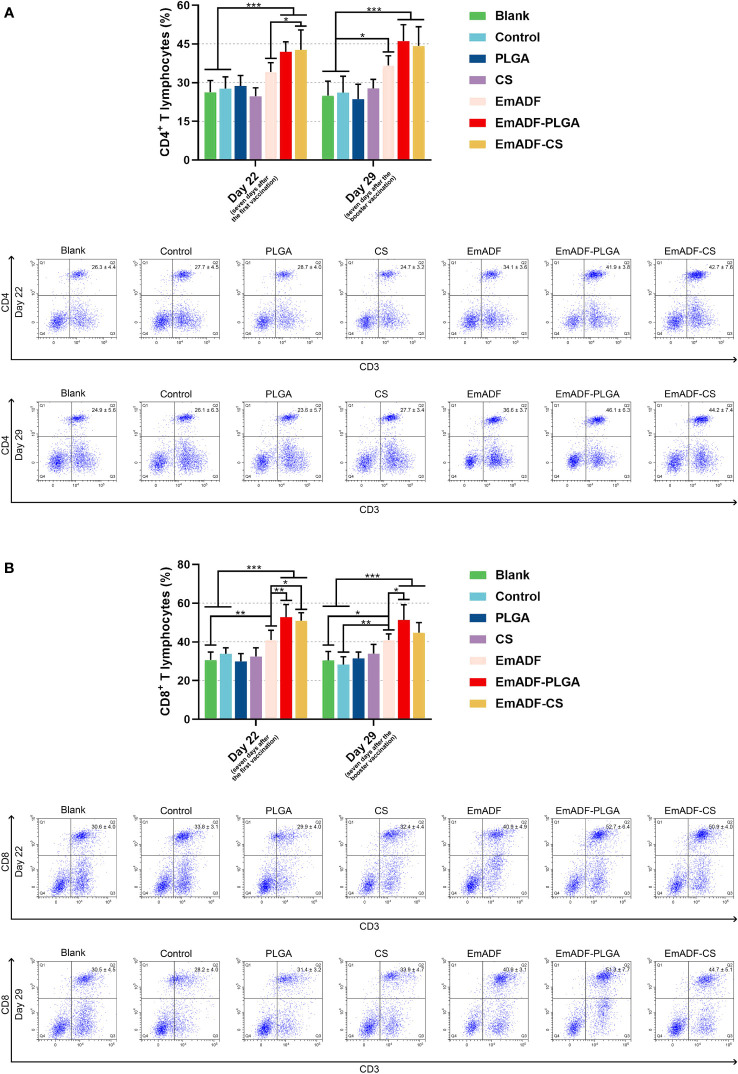
Flow cytometry analysis of CD4^+^ **(A)** and CD8^+^ T lymphocytes **(B)** in splenocytes at the age of 22 and 29 day. Chickens in each group were sacrificed, and the lymphocytes was collected. Adequate compensation was conducted before cell sorting, and the lymphocytes were analyzed by flow cytometry. Each group involved five biological replicates, and each replication was detected once. Values were estimated using one-way ANOVA analysis followed by Dunnett’s test. Comparisons among EmADF, EmADF-PLGA, and EmADF-CS group were conducted by ANOVA following Bonferroni’s correction. Values were presented as the mean of the mean ± SD. *p< 0.05, **p< 0.01, and ***p< 0.001.

Seven days after the first and booster vaccination (at age of 22 and 29 day), lymphocytes were separated by using the previously described method. After incubation with the antibodies, lymphocytes were sorted by a flow cytometry. As evaluated in **Figure 8A**, EmADF-PLGA and EmADF-CS nanospheres could induce significantly higher proportions of CD4^+^ T cells at the age of 22 (seven days after the first immunization) and 29 day (seven days after the booster immunization, *p*< 0.001). As for the CD8^+^ T cells showed in **Figure 8B**, rEmADF and its encapsulations could statistically increase the expression of CD8 molecules on the surface of CD3^+^ T lymphocytes after the first and second immunizations. Furthermore, EmADF-PLGA nanospheres exhibited the capacity in inducing the differentiation of lymphocytes into CD8^+^ T cells when compared with rEmADF at the age of 22 (seven days after the first immunization) and 29 day (seven days after the booster immunization, *p*< 0.05). It is noteworthy that no significance was detected in the lymphocytes across any negative group (*p* > 0.05).

### 3.6 Weight analysis and protective efficacy

**Figure 9:**
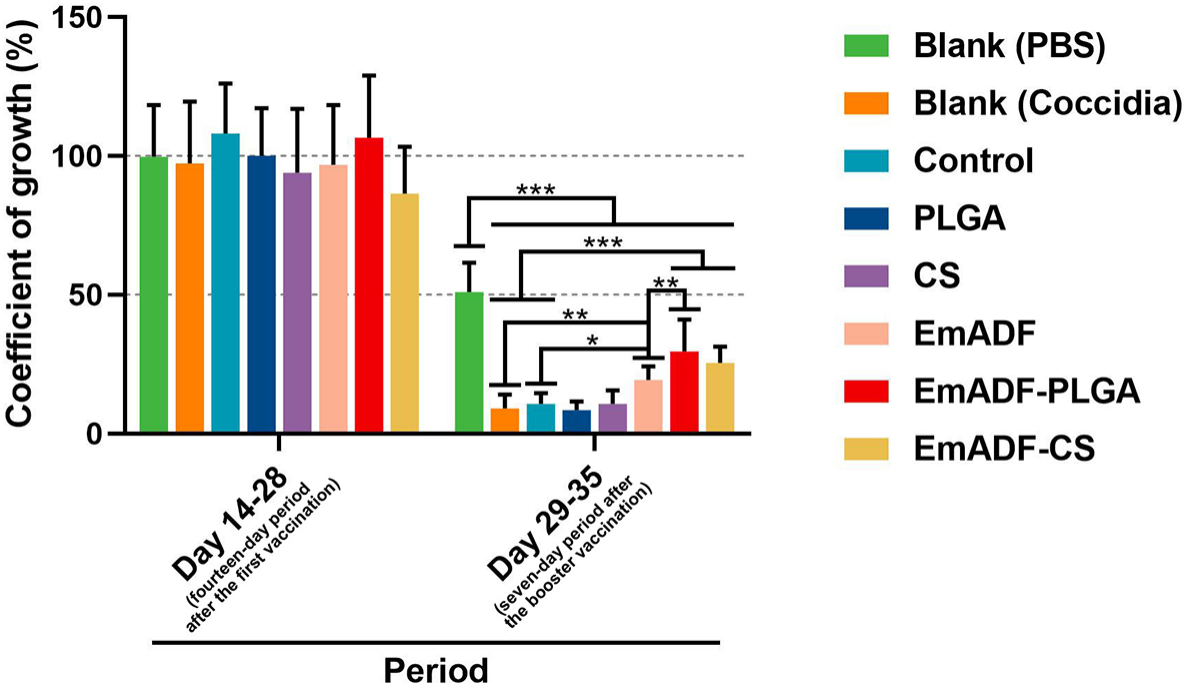
The coefficient of growth in immunized chickens. Each chicken was orally challenged with 5 × 10^4^ purified *E. mitis* oocysts seven days after the second vaccination. Chickens were weighted at the age of 14, 28, and 35 days, and the coefficient of growth was calculated. Each group involved ten biological replicates, and each replication was detected once. Values were estimated using one-way ANOVA analysis followed by Dunnett’s test. Comparisons among EmADF, EmADF-PLGA, and EmADF-CS group were conducted by ANOVA following Bonferroni’s correction. Values were presented as the mean of the mean ± SD. *p< 0.05, **p< 0.01, and ***p< 0.001.

In order to study the synthesized nanospheres on weight changes of infected animals, ten chickens from each group were high-dose challenged. All chickens were weighted at the age of 14 (before the first immunization), 28 (seven days after the first immunization), and 35 day (seven days after the booster immunization), and the growth efficiency was calculated. Furthermore, all infected chickens survived during the experimental period. As illustrated in **Figure 9**, all challenged chickens exhibited significantly inhibition in growth efficiency as compared to the chickens challenged with 1 × PBS (50.94 ± 10.63%, *p*< 0.001). When compared with the challenged animals in negative group (9.11 ± 4.93% in Blank (Coccidia) group, while 10.64 ± 3.86% in control group), animals immunized with rEmADF-loaded nanospheres (19.28 ± 4.9%) showed higher growth efficiency (*p*< 0.001). In addition, among all challenged groups, EmADF-PLGA group (29.56 ± 11.46%) generated significantly higher growth efficiency than EmADF group (25.48 ± 5.84%, *p*< 0.01), while no significant difference evaluated in rEmADF-loaded nanoshperes (*p* > 0.05). All these obtained results highlight the significance of EmADF-PLGA nanospheres in up-regulating the growth efficiency after the infections of *E. mitis*.

**Figure 10:**
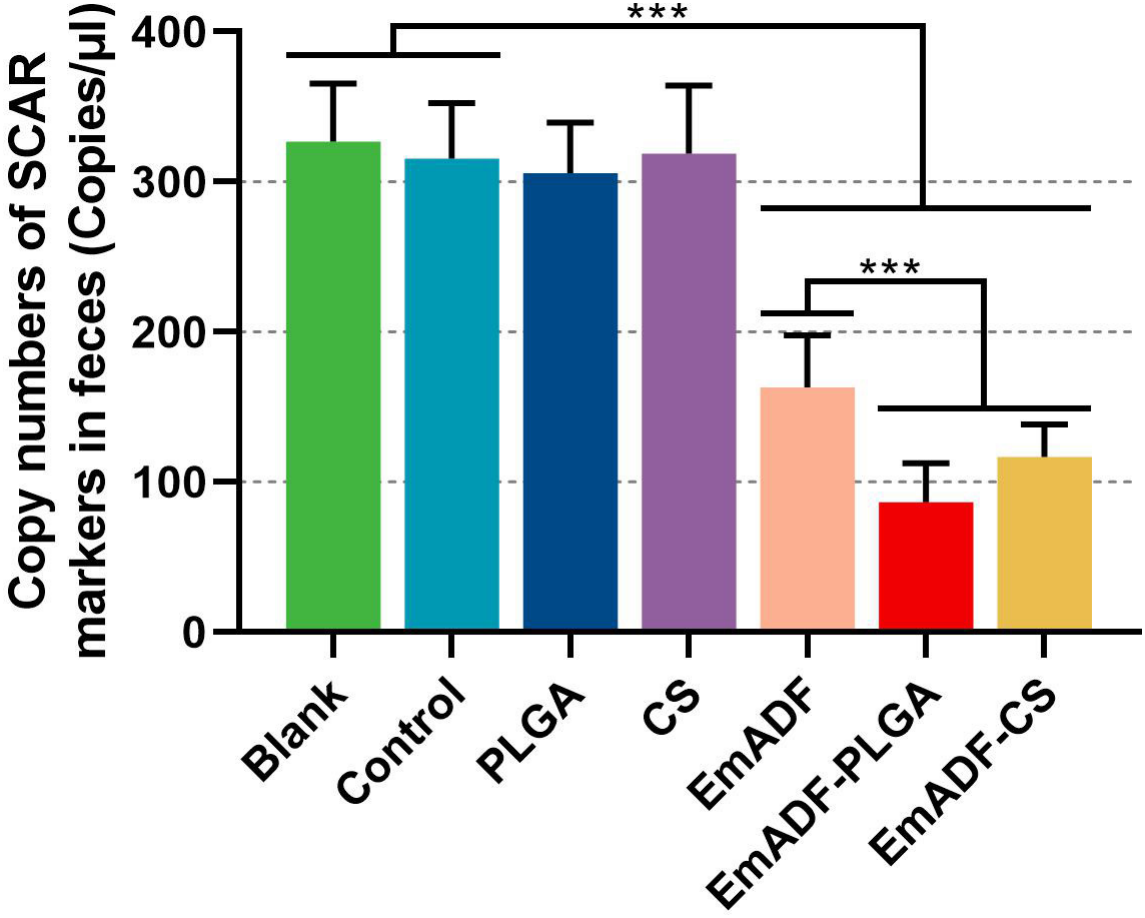
*E. mitis* oocyst burdens in the excreted feces from low-dose challenged chickens. Each chicken was orally challenged with 3,000 purified *E. mitis* oocysts seven days after the booster immunization. Seven days after challenge, feces of each chicken were collected for DNA extractions. Each group involved ten biological replicates, and each replication was detected three times. Values were estimated using one-way ANOVA analysis followed by Dunnett’s test. Comparisons among EmADF, EmADF-PLGA, and EmADF-CS group were conducted by ANOVA following Bonferroni’s correction. Values were presented as the mean of the mean ± SD. ***p< 0.001.

To investigate the immunoprotection of the prepared nanospheres, chickens were low-dose challenged. All vaccinated animals survived after *E. mitis* infections, and the oocyst burdens of each chickens were analyzed by qPCR seven days after challenge. When compared with the blank (326.80 ± 38.69 copies) or control group (315.60 ± 36.82 copies), significantly inhabited levels of SCAR markers were evaluated in EmADF (163.07 ± 34.69 copies), EmADF-PLGA (86.52 ± 26.03 copies), and EmADF-CS groups (116.98 ± 21.51 copies, *p*< 0.001, **Figure 10**), indicating rEmADF enhanced host immunity against *E. mitis*. Moreover, compared with chickens in EmADF group, chickens in EmADF-PLGA (*p*< 0.001) and EmADF-CS group (*p*< 0.001) were detected with lower burdens of *E. mitis*, emphasizing nano-material could boost the immune response. Noticeably, EmADF-PLGA nanospheres showed fewest parasite burdens in the collected feces, demonstrating the strongest anti-*E. mitis* effect. All these findings suggested the efficiency of synthesized nanospheres in eliciting stronger immunity against *E. mitis*.

## 4 Discussion

As the intracellular protozoan parasite, individual or several *Eimeria* species could cause intestinal coccidiosis in chickens (2, 44), and cause huge economic losses to the poultry industry (12, 45). Compared with medical treatment, poultry immunization has been thought to be critical in reducing occurrence of coccidiosis (15). In recent years, minimal components from *Eimeria* spp. are used as a novel approach to elicit avian coccidiosis resistance (15). Such approaches are designed according to parasites’ antigens, and the immunogenicity of parasites remained (46). Detailed studies in *Eimeria* genomics with novel tools have revealed many microbial proteins that are considered as the candidates of vaccine antigens (47, 48). Many *Eimeria* antigens are utilized to construct the anticoccidial vaccines, such as surface antigen (49), apical membrane antigen (11, 50), microneme (51, 52), rhoptry, profilin (53) etc. Although the antigens mentioned above have been proved to be effective in eliciting host immunity against the *Eimeria* species, these antigens could not provide fully immunoprotections against coccidiosis. In addition, different antigens could induce *E. mitis*-specific immunity through different immune effector mechanisms, and the immune effects were mainly evaluated by parasite-challenge (14). The present study focused on the *E. mitis* actin-depolymerizing factor, which intimately participated in the modification of the cytoskeleton and motility of *Eimeria* species (54). The recombinant EmADF protein was first expressed by the prokaryotic expression system, and the immunogenicity was investigated by western blot. Then rEmADF was entrapped in nanospheres to protect protease-antigens from degradation, and the immunoprotection of synthesized nanospheres was subsequently analyzed in animals. The results indicated that EmADF-PLGA and EmADF-CS nanospheres were spherical in shape and nontoxic with satisfactory immunogenicity. *In vivo*, rEmADF-loaded nanospheres exhibited potent immune enhancement in inducing humoral and cellular immune response, and were capable of evoking growth efficiency and inhabiting *E. mitis* burdens in feces. All these obtained findings suggested that EmADF-PLGA and EmADF-CS nanospheres could be an efficient approach to prevent the infections of *E. mitis*.

Nanosphere-based vaccines offer the chances as highly safe and effective alternatives to traditional subunit vaccines (55, 56). Currently, many techniques have been exhibited much advantage in nanosphere formulation (40, 57). Under such circumstances, we successfully prepared rEmADF-loaded PLGA and CS nanospheres by double emulsion solvent evaporation and ionic gelation technique, respectively. The obtained EmADF-PLGA and EmADF-CS nanospheres were nanosized with spherical shape that was considered as easier to be absorbed by cells (58). As reported previously (59), nanospheres sized approximately 100 nm were easier to go through cell membrane in Hela cell lines when compared with those sized about 1,000 nm in diameter. The published studies also showed that the CS nanospheres sized about 300 nm in diameter gained better absorption compared with the nanospheres sized 1,000 and 3,000 nm in diameter (60). All these results imply that our prepared nanospheres may have more advantages in inducting host immunity. Notably, LC and EE of nanospheres vary in the published studies. Followed by the similar procedures, the EE and LC reached 82.40 ± 0.06% and 2.00 ± 0.01% respectively in PLGA-rEtTA4 nanospheres (40). Reported in another paper, the EE was 89.35 ± 1.18% in PLGA-rEm14-3-3 nanospheres, and in CS-rEm14-3-3 nanospheres reaches 83.46% (61). However, by using similar procedures with minor modifications, the EE reached 55% in the formulation of CS-PLGA-rOmp22 nanospheres, while the LC was about 0.94% (62). Such phenomena may be caused by the encapsulated antigens and synthesized methods, and furthermore affect LC and EE (36, 57). The subsequent studies should focus on the effects of loaded antigens and synthesized procedures on LC and EE in formulating nanospheres.

Due to the slow diffusion of the loaded antigens into the medium, nanospheres can decrease the times of immunity and improve the antigen-presenting in APCs (63, 64). In the current research, the slow-release profile of EmADF-PLGA and EmADF-CS nanospheres was observed, and a more stable release was also detected in EmADF-CS nanospheres when compared with the EmADF-PLGA nanospheres. Moreover, the synthesized nanospheres were spherical in appearance, which seems play an important role in inhabiting obvious burst release. As a polyester that is nontoxic, PLGA could combine with lipid monolayers, and then promote the slow release of antigens (64). Similarly, chitosan, served as a cation polysaccharide, can bind to the cell membranes, leading to a long-term residence (65). However, the burst release of formulated nanospheres occurred at the first day, and such characteristic may be driven by the unbound antigens. In addition, the diameter, polarity, molecular weight, even the encapsulations of the nanospheres can affect the burst release (66). Noticeably, the prospect of nanospheres in vaccines is usually limited by their toxicity for mammals (67). In all reagents used in nanosphere formulations, only DCM was regarded as toxic and hard to erase by evaporation (68). Therefore, the EmADF-PLGA nanospheres were fully freeze-fried to fully remove the toxicity. Unsurprisingly, no toxic side effect occurred and all experimental animals were kept in good clinical status, demonstrating the synthesized nanospheres could be applied to animal immunization.

By preventing the parasites from attaching to the surface of host cells (69), largely produced IgY plays an crucial role in resisting the infection of *Eimeria* species (70). Based on the results of standard ELISA, high titers of EmADF-specific IgY antibody were illustrated in chickens vaccinated with rEmADF-loaded nanospheres. These findings implied the qualified immunogenicity of expressed rEmADF, and the enhanced humoral immunity induced by the rEmADF-loaded nanospheres.

Cytokines are the essential factors in modulating naïve T cells differentiation into either Th1 or Th2 type cells (71, 72), and play a crucial role in the process against avian coccidiosis (1, 8). By augmenting the production of IFN-γ, the pro-inflammatory cytokines enhance the Th1 immune response, which is considered to be predominant in resisting the replications of *Eimeria* species (72–74). Based on the results of double antibody sandwich ELISA, promoted levels of IFN-γ were confirmed in animals’ sera vaccinated with EmADF-PLGA and EmADF-CS nanospheres, emphasizing the Th1-related immune response was induced. Additionally, driven by IL-4 cytokine, Th2 immunity also plays an important role in resisting the coccidiosis (72). Secreted by the CD4^+^ follicular helper T (Tfh) cells and CD4^+^ Th2 cells (75), IL-4 cytokines mediate humoral immunity in the interface of host and parasite (76). Significant high levels of IL-4 cytokine were evaluated in the animals vaccinated with rEmADF and its nanospheres in the current study, demonstrating the Th2 cell mediated and Tfh-related immunity was activated. With the existence of TGF-β, CD4^+^ Th2 cells can differentiate to CD4^+^ Th9 cells as previously reported (77, 78). Participated in the immunity against *Eimeria* species, the activated CD4^+^ Th9 cells could release IL-10, an anti-inflammatory cytokine, which related to the maintenance and reestablishment of host immune system (79). However, as a double-edged sword largely released by inducible regulatory T (iTreg) cells, TGF-β cytokines are mainly involved in host immunosuppression (80). At the present study, animals vaccinated with rEmADF-loaded nanospheres were observed with enhanced TGF-β and IL-10 at the age of 29 day, indicating the Th9 cell mediated immunity was activated in host immune response against avian coccidiosis, and the slight immunosuppression was also induced. By inducing the specific differentiation of naïve T cells to CD4^+^ Th17 cells (81), IL-6 cytokines play an important role in connecting innate to the adaptive immunity (82). In addition, IL-6 cytokines are also proved to be essential in induction of cytotoxic T cells (83). Morevoer, the mRNA levels of chicken IL-17 cytokines are up-regulated in intestinal intraepithelial lymphocytes (IELs) after *Eimeria* infections, suggesting the IL-17 cytokine is related to the immunity against the invasion of parasites (84, 85). As a symbolic cytokine generated by CD4^+^ Th17 cells, IL-17 also participated in the secretion of IL-6 (86), and exerts a pro-inflammatory effect in inhabiting the infections of *Eimeria* species (87, 88). In our study, the secretions of IL-6 and IL-17 were statistically promoted in the animals vaccinated with rEmADF and its nanospheres, indicating the adaptive immune response was induced in anti-*E. mitis* defense.

With the participation of both CD4^+^ and CD8^+^ T lymphocytes, cell-mediated immunity exhibits a dominant role in inducing an antigen-specific immunoprotection against *Eimeria* species (1, 72, 89). Acted as the effector cells, CD8^+^ T cells could generate cytokines to generate cytotoxic effect in anticoccidial immunity, while CD4^+^ T cells were of great assistance in the formulation of CD8^+^ T cells (72, 76). Furthermore, the activation of CD4^+^ T cells requires two signals, major histocompatibility complex II (MHC-II) and costimulatory molecules (90, 91), while CD4^+^ T cells as well as the antigen-presenting cells (APC) play an important role in CD8^+^ T cell activation (92, 93). The activated CD8^+^ T cells will go through two phases: proliferation and differentiation (94). Different Th lymphocytes can be polarized by T lymphocytes, and determine the type of host immunoprotection (95). In the current study, CCK-8 assay was recruited to illustrate the effects of rEmADF on the lymphocyte proliferation *in vivo*. Compared with the rEmADF-immunized group, groups immunized with rEmADF-loaded nanospheres displayed greater proliferation after the booster immunization, suggesting EmADF-PLGA and EmADF-CS nanospheres were essential in promoting the proliferation of splenic lymphocytes in chicken. In addition, the flow cytometry was recruited to evaluate the proportions of CD4^+^ and CD8^+^ T cells, and all positive groups were illustrated with higher levels compared with the negative groups, indicating that rEmADF as well as its encapsulations were mainly responsible for the percentages of CD4^+^ and CD8^+^ T cells in animals. According to results, rEmADF and its encapsulations could induce the generation of cellular immunity against coccidiosis, and the encapsulations in nanospheres could further confer the cellular resistance.

Many researches indicate that encapsulations in nanospheres could strengthen the entrapped antigens in host immunity against specific pathogens (96). Entrapped in a novel adjuvant, named QCDC, the recombinant *E. acervulina* profilin proteins could better up-regulate weight gains and reduce the oocysts in feces excreted by the challenged animals (97). Encapsulated in the nanospheres formulated by Xu et al., *E. mitis* 1a protein exhibited higher immune response in resisting parasites (98). Similar findings also confirmed by Huang et al. in Hy-Line chickens vaccinated with PLGA nanospheres loaded with recombinant *E. tenella* TA4 proteins (40). To analyze the best encapsulation in the current study, the weight changes and parasites burdens of immunized chickens after challenge were investigated. Chickens were high-dose challenged to evaluate the impact of synthesized nanospheres on animals’ growth efficiency, and only the EmADF-PLGA nanospheres induced statistically higher growth efficiency compared with the naked antigens. To demonstrate the effects of nanospheres in inhabiting *E. mitis* oocysts, animals were low-dose challenged and the copy number of SCAR markers in feces were investigated by qPCR. The results showed chickens immunized with EmADF-PLGA or EmADF-CS nanospheres received a lower parasites burden in feces. Highlighted its advantages in promoting the growth efficiency and resisting the infections of *E. mitis*, all these obtained findings lent credit to the idea that both EmADF-PLGA and EmADF-CS nanospheres could induce a satisfactorily protective immunity.

## Conclusion

Collectively, our research first prepared rEmADF by prokaryotic expression. Entrapped in PLGA and CS nanospheres *via* double emulsion solvent evaporation technique and ionic technique, the protective efficiency of rEmADF and its encapsulations were then evaluated in chickens. The obtained results ratified that rEmADF-loaded nanospheres could elicit strongly humoral and cellular immunity against the infections of *E. mitis*. Compared with naked antigens, EmADF-PLGA and EmADF-CS nanospheres could significantly increase host immunity against *E. mitis*, and were regarded as the superior vaccines in the present research. Despite its strongly protective efficiency, both EmADF-PLGA and EmADF-CS nanospheres could not provide fully protections against the infections of *E. mitis*. Consequently, future studies on the novel nanospheres should optimize the administration strategy, dose, and route to enhance host immune protections as well as the animals’ growth efficiency.

## Data availability statement

The original contributions presented in the study are included in the article/**Supplementary Material**. Further inquiries can be directed to the corresponding author.

## Ethics statement

The animal study was reviewed and approved by Animal Ethics Committee, Nanjing Agriculture University, Nanjing, China.

## Author contributions

LX and XL designed the research. ZY, LX and KH conducted the research. ZY, KH, and ML analyzed the data. ZY and LX wrote the manuscript. RY and XS participated in the revision of the manuscript. All authors contributed to the article and approved the submitted version.
