# Supplementary material for: Actin depolymerizing factor-based nanomaterials: A novel strategy to enhance E. mitis-specific immunity
Source: Front Immunol. 2022 Dec 21;13:1080630. doi: 10.3389/fimmu.2022.1080630 (PMC9810622; doi:10.3389/fimmu.2022.1080630)
Supplement: Supplementary file 1 [file DataSheet_1.docx]

Supplementary Material


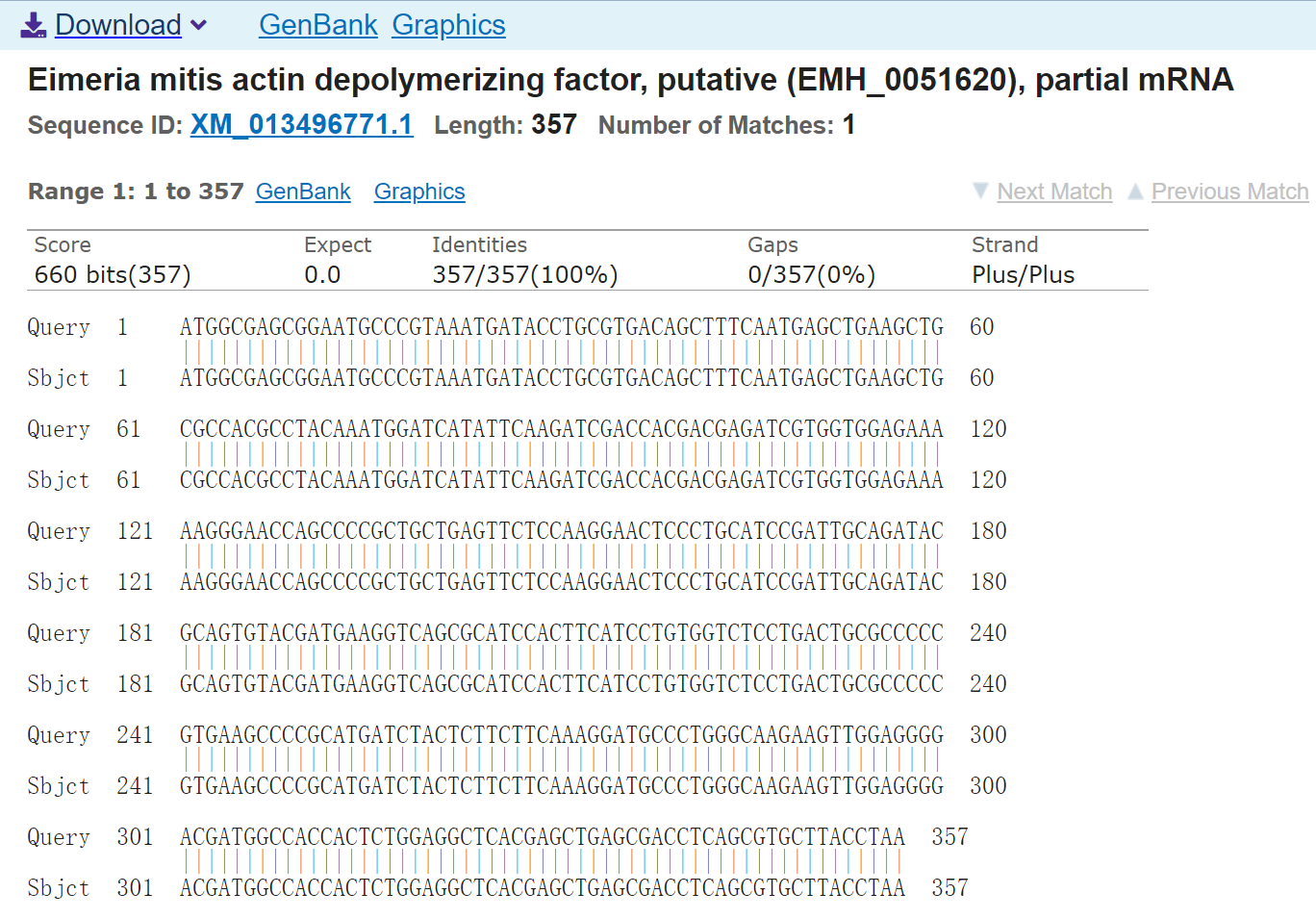


**Figure S1.** Sequence alignment results of the pET-32a-EmADF vector. Compared with the nucleotide sequences of *E. mitis* ADF gene (Genbank: XM_013496771.1), sequence analysis was carried out by the online Blast program (https://blast.ncbi.nlm.nih.gov/Blast.cgi).
